# Gluteal compartment syndrome following alcohol intoxication: Case report and literature review

**DOI:** 10.1016/j.amsu.2019.07.010

**Published:** 2019-07-11

**Authors:** Adel Elkbuli, Carol Sanchez, Shaikh Hai, Mark McKenney, Dessy Boneva

**Affiliations:** a Department of Surgery, Kendall Regional Medical Center, Miami, FL, USA; b University of South Florida, Tampa, FL, USA

**Keywords:** Gluteal compartment syndrome, Acute compartment syndrome, Rhabdomyolysis, Fascial compartments

## Abstract

**Introduction:**

A compartment syndrome (CS) occurs when increased pressure within an anatomic compartment leads to inadequate perfusion. Although rare, gluteal CS can be encountered when an unconscious person has a prolonged period of immobilization.

**Presentation of case:**

A 20-year-old male with history of polysubstance abuse leading to passing out, presented with right buttock and lower extremity pain, increased creatinine phosphokinase (CPK), and acute renal failure. Physical examination and MRI confirmation supported gluteal CS. Patient was taken to the OR for gluteal fasciotomy. Afterwards, the pain improved, the CPK and creatinine trended to normal. He was discharged home on day 7.

**Discussion:**

CS can occur in any part of the body with fascial compartments. Increased compartmental pressure causes compression of vessels and nerves in the area that can lead to ischemia and necrosis. CS can occur after trauma, excessive fluid resuscitation, or surgery. It is also reported due to the prolonged periods of immobilization and increasing pressure on dependent areas. Often, intra-compartmental pressure is measured to confirm the diagnosis. The mainstay of treatment is fasciotomy.

**Conclusion:**

Due to the rarity of gluteal compartment syndrome, the diagnosis is often delayed. If the affected area is ischemic for a significant amount of time, it can lead to sciatic nerve palsy, paresthesias, paralysis and muscle necrosis. Patients may experience irreversible damage after the syndrome and as such providers should be cognizant of this clinical entity to make an early diagnosis of gluteal compartment syndrome.

## Highlights

• Gluteal compartment syndrome is rare and a high index of suspicion is needed to treat in a patient with a typical history.
• If recognized early this is relatively easy to treat with minimal untoward sequelae or loss of function.
• If the fasciotomy is delayed, ischemia can cause irreversible muscle damage and functional loss.

## 1 Introduction

**Fig. 1:**
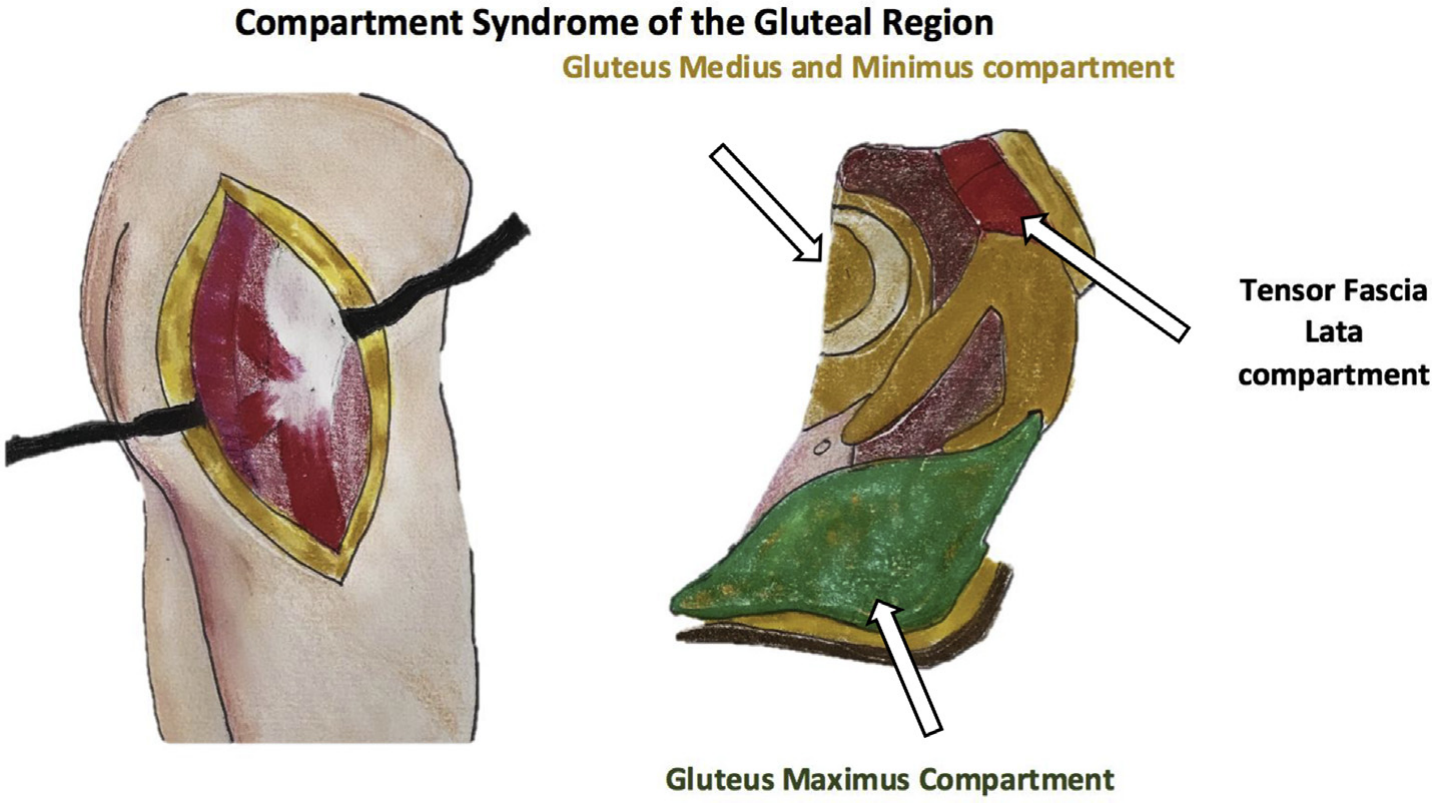
Anatomy of the gluteal compartments.

Acute compartment syndrome (CS) results from increased pressure in a closed anatomic fascial area, which can lead to reduced perfusion [1]. The expansive force, usually of edema, in the affected area leads to increased venous pressure, venous occlusion and eventually decreased arterial circulation and if continued unabated, resulting in ischemia and necrosis. Discrete fascial compartments exist throughout the body with the most common compartment affected being the anterior compartment of the infra-geniculate lower extremity. However, acute compartment syndrome is also seen in the thighs, forearm, buttock, shoulder, hand, and foot. Fig. 1 shows the anatomy of the gluteal compartment. The gluteal compartment is made up by three non-distensible fascial compartments: 1) tensor fascia lata compartment, 2) gluteus medius and minimus compartment, and 3) gluteus maximus compartment**.** The tensor fascia lata compartment is anterior and is comprised by the tensor fascia lata muscle. The gluteus medius/minimus compartment holds the gluteus medius and minimus muscles in a compartment between the tensor fascia lata and gluteus maximus. The gluteus medius being the most lateral of the two muscles in its compartment. Finally, most posterior compartment holds the gluteus maximus muscle [2]. A CS is typically associated with fractures, most commonly a tibial fracture or a distal radius fracture. Other associated events leading to a CS include drug overdose, vascular injuries, burns, bleeding disorders, excessive fluid resuscitation and reperfusion injuries.

Concerning gender prevalence, young males (<35 years) are 10 times more likely to suffer from this and typically more affected than females [1]. The average annual incidence is estimated to be about 1–7.3/100,000. The pathophysiology of this syndrome is essentially due to increased intra-compartmental pressure that eventually leads to impairment and reduction of first lympho-venous and then eventually arterial blood flow that results in ischemia. This is called the arterio-venous gradient theory and a vicious cycle is set up as slow venous drainage leads to increased third spacing of fluid in the interstitium exacerbating the ischemia. As mentioned, if the ischemia is severe, there is risk for irreversible muscle necrosis. Normal physiological levels of intra-compartmental pressure are <10 mmHg, however the exact values are dependent on the patient's perfusion pressure and directly correlated with the mean arterial perfusion pressure. Once this is increased to >30 mmHg, or approaches acute CS should be suspected [1]. However, a normal intra-compartmental pressure does not rule out the presence of CS. Prolonged increased pressure in these compartments can lead to compression, low nutrient delivery and oxygen tension. Prognosis tends to be optimal for those patients who have a fasciotomy performed within 6 hours. This disease can even prove to be fatal if significant delays to treatment are encountered and cause infection of the compartment, muscle necrosis and acute renal failure [1]. This case has been reported in line with the SCARE criteria [3].

### 1.1 Presentation of Case

**Fig. 2:**
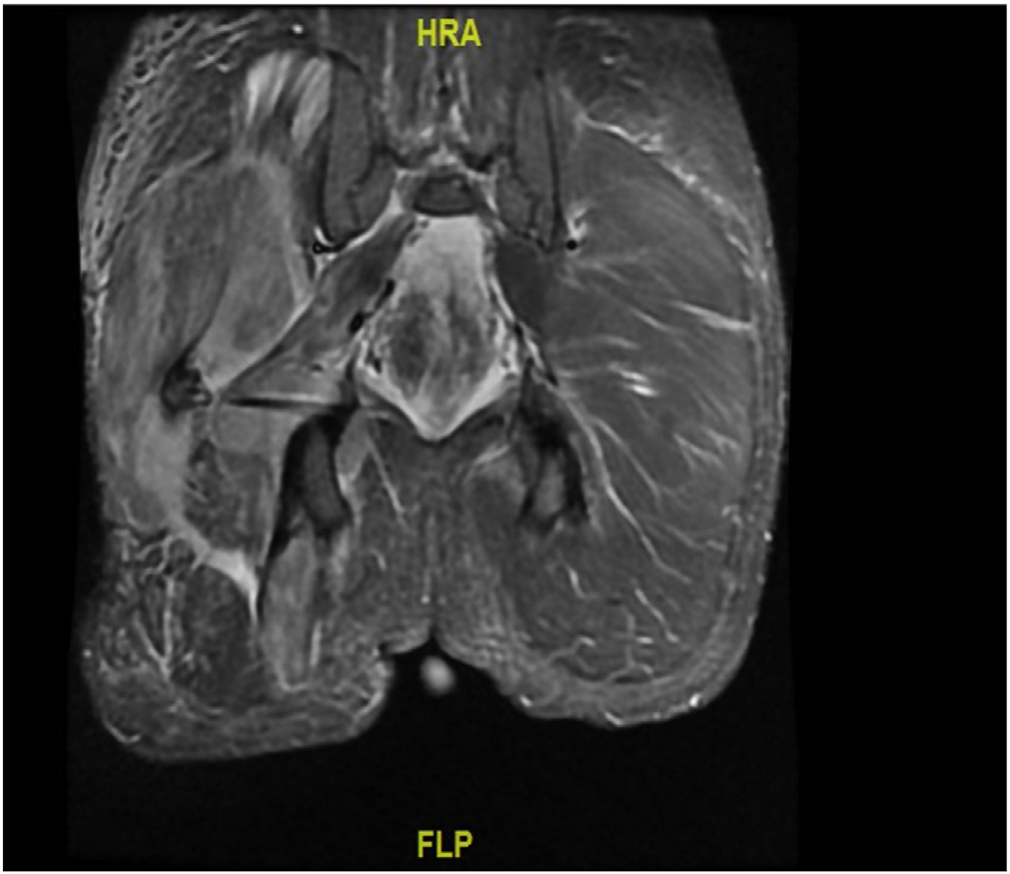
MRI Showing Right hip musculature and intermuscular fascial edema in the right hip: characteristics for post-traumatic changes.

A 20-year-old white male presented to the Emergency with a history of polysubstance abuse-marijuana, benzodiazepines, and heavy chronic alcohol intake. His main complaint was right buttock pain extending down his thigh. Patient reported passed out on his right side and had no specific trauma to the area. Physical exam revealed limited and painful range of motion of the right leg with a tense gluteal region. The patient's creatinine phosphokinase (CPK) levels on admission were over 60,000 (normal = 15–105 u/L) and creatinine was 2.66 mg/dL (normal 0.6–1.22 mg/dL. MRI of the right hip showed extensive muscular edema affecting the right adductor and abductor musculature, which was consistent with rhabdomyolysis (Fig. 2).

**Fig. 3:**
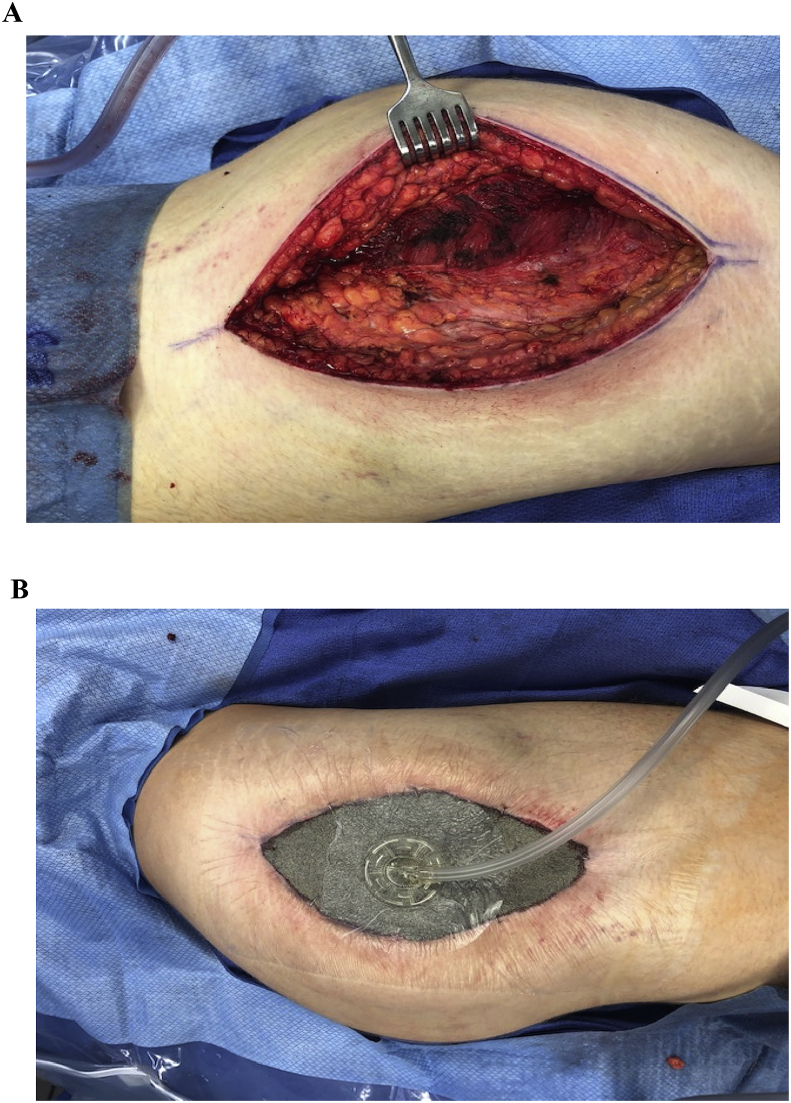
**A.** Fasciotomy incision of the gluteal region; the patient is in the left lateral decubitus position. A right lateral incision (modified-Gibson approach) was utilized. The head of the patient is to the left of the picture. **B.** Temporary vacuum dressing placed over gluteal incision.

Patient was transferred to operating theatre for decompressive fasciotomy of the gluteal area for diagnosis of gluteal CS. Decompressive fasciotomy of gluteus maximus, minimus, and tensor fascia lata compartments relieved all pressure and muscle was free to bulge out through the compartments (Fig. 3A). Vacuum Assisted Closure (VAC) device was placed on the patient for wound management (Fig. 3B). CPK and creatinine levels trended to normal after treatment with intravenous (IV) fluids with bicarbonate. Additionally, he was placed on chlordiazepoxide taper for prevention of delirium tremens and alcohol withdrawal. Four days later, he returned to the OR for primary closure of fasciotomy site. Smoking, substance abuse and alcohol cessation advice and treatment options were rendered. The patient was discharged on hospital day 7 in stable condition and follow up 2 weeks later in the office showed the patient to be doing well.

## 2 Discussion

Gluteal CS is rare and often associated with prolonged immobilization and direct pressure after drug abuse [4]. Other causes of gluteal CS involve immobilization during/after surgeries like aortic dissection repairs, bariatric surgery and direct trauma [[5], [6], [7]]. Sarwar et al. describe the case in which a patient presented with gluteal CS following microsurgery to the hand, which had never been documented. This lends itself as evidence for the pathophysiology behind prolonged periods of vessel compression and its association with gluteal CS [8]. Similar findings were present in a case of a 65-year old who had been reported unconscious after heroin use. Due to the extended period of unconsciousness, the patient presented with symptoms of CS not only in the right gluteal region, but also in the right upper arm, which is also a very rare finding. Reported symptoms and physical exam findings were typical of CS with edema and pain in affected sites. Like our present patient, this case reported polysubstance abuse [9].

Classically 5 “P” are associated with this CS diagnosis. Pain, Paresthesia, Pallor, Paralysis and increased intra-compartmental Pressure. Physical exam findings can reveal edema, tenderness, and erythema surrounding the affected tissue. Patients can also present initially with tingling sensation and paresthesias and then progress to severe pain on passive motion disproportionate to the injury. In the buttocks region it is associated paresthesia and limited range of motion [10]. These physical exam findings are consistent with the present case with the exclusion of the paresthesia, which the current patient did not report. Beyond pain and paresthesia, physical exam findings can be related to a neuropraxic sciatic nerve. Although the sciatic nerve does not lie within the gluteal compartment, it does lie posterior to the gluteus maximus. Therefore, if there is increased swelling of this muscle, it can result in compression of the sciatic nerve and sciatic nerve palsy [10]**.** Sciatic nerve palsy is characterized by pain, numbness, burning, and tingling along the distribution of the sciatic nerve. It can also present as weakness of the muscles innervating the peroneal muscles. Our patient's lack of complaints of paresthesia indicate possible non-compression of sciatic nerve due to the gluteal CS. Sciatica has been reported in other cases of gluteal CS with good prognosis following fasciotomy. As the muscle is allowed to bulge out, the pressure decreases and the nerve becomes decompressed with reversal of symptoms [11]. Although resolved in our patient, sciatic nerve palsy can become irreversible and present even during one-year follow up as was reported by Dilernia et al. The 12-h delay in transfer to their hospital could be an explanation for persistent sequelae after the syndrome. Irreversible sciatic nerve palsy was also reported in an additional case report, where neurologic symptoms and back pain were still present one year later [12]**.** Treatment with fasciotomy is recommended for normotensive patients with intra-compartmental pressures >30 mmHg. In hypotensive patients, only 20 mmHg may be enough to cause signs of increased compartment pressures. Some researchers state that a more accurate way to diagnose CS is to measure the intracompartmental pressure and note if its (delta pressure) within 30 mmHg of the patients diastolic pressure [13]. The pressure was not measured in the present patient due to historical and clinical evidence highly suggestive of gluteal CS. Clinical findings such as increased levels of creatinine phosphokinase and increased creatinine, coupled with historical components of drug abuse and paresthesia, were sufficient diagnose this patient with gluteal CS [14]. Measurement of compartment pressures can be done on patients who are unconscious and whose clinical and historical components cannot be appropriately assessed. Prior studies have reported the time-sensitive treatment of ischemia such that six-eight hours of muscle ischemia is often irreversible [14]**.**

Substance abuse is an independent risk factor for non-traumatic, non-exertional rhabdomyolysis through mechanisms of imbalanced electrolyte levels, dehydration, malnutrition and more. Additional risk of rhabdomyolysis in this population is conferred through high risk of CS in unconscious states. It would be expected that rhabdomyolysis exacerbations would be seen in these patients. Previously, this has been associated with acute kidney injury due to damaging effects of myoglobin released from the myocytes and depositing in to the renal tubules [15]. This patient presentation reiterates the finding that, although very rare in occurrence, patient's outcomes are heavily reliant on keeping CS as a differential. Specifically, in substance abuse populations, doing so can help prevent more severe sequelae such as acute kidney injury from manifesting and potentially leading to irreversible damage if there are delays in the treatment and management of CS.

## 3 Conclusion

Gluteal compartment syndrome can occur after periods of prolonged immobilization and pressure in a localized area. Clinical diagnosis with elevated CPK and creatinine should be considered for early treatment with 3-compartment gluteal fasciotomy to prevent further complications such as sciatic nerve palsy, kidney damage, and even death.

## Ethical approval

This case report was conducted in compliance with ethical standards. Informed written consent has been obtained and all identifying information is omitted.

## Sources of funding

None.

## Author contribution

Adel Elkbuli, Carol Sanchez, Dessy Boneva, Mark McKenney– Conception of study, acquisition of data, analysis and interpretation of data.

Adel Elkbuli, Dessy Boneva, Carol Sanchez, Shaikh Hai, Mark McKenney - Drafting the article.

Dessy Boneva, Mark McKenney – Management of case.

Adel Elkbuli, Carol Sanchez, Dessy Boneva, Shaikh Hai, Mark McKenney – Critical revision of article and final approval of the version to be submitted.

## Conflicts of interest

None.

## Trial registry number – ISRCTN

NA.

## Guarantor

Dessy Boneva.

Mark McKenney.

## Research registration Unique Identifying Number (UIN)

This is a case report study.

## Provenance and peer review

Not commissioned, internally reviewed.

## Informed consent

Patient informed written consent was obtained and all identifying information is omitted.

## Appendix A Supplementary data

Scare Checklist Scare Checklist
